# Type 1 and type 2 torpedo maculopathy

**DOI:** 10.1007/s00417-024-06386-0

**Published:** 2024-01-30

**Authors:** Annekatrin Rickmann, Jan-Philipp Bodenbender, Faik Gelisken, Laura Kühlewein

**Affiliations:** 1 Knappschaft Hospital Saar, Department for Ophthalmology, Sulzbach, Germany; 2 University Eye Hospital, Department for Ophthalmology, Eberhard Karls University, Tübingen, Germany

**Keywords:** Torpedo maculopathy, Torpedo retinopathy, Choroidal excavation, Fundus autofluorescence, Optical coherence tomography

## Abstract

**Purpose:**

To analyze torpedo maculopathy (TM) and to report the characteristics of the disease.

**Methods:**

Retrospective study. The review of a database for clinical diagnosis identified eight patients with TM lesions in the retina between 2016 and 2022. Multimodal imaging was used to analyze the cases.

**Results:**

All cases were unilateral, asymptomatic, and hypopigmented. They were associated by surrounding hyperpigmented retinal pigment epithelium changes to varying degrees. All lesions were located in the temporal retina on the horizontal axis, pointing towards the fovea, except for one patient with a lesion inferior to the fovea. Optical coherence tomography imaging revealed a normal inner retina in all eyes. In the area of the TM lesion, attenuation of the interdigitation zone was seen in mild cases (three cases). All other five patients had thinning of the outer nuclear layer and loss of ellipsoid zone and interdigitation zone of the TM lesion. Four of these cases had a subretinal cavitation/cleft, and two of them additionally an inner choroidal excavation. No patient had any sign of choroidal neovascularization. The average age for patients with type 1 TM was 18 years and for type 2 TM 16.5 years.

**Conclusion:**

In this large case series, we could not detect an age difference between the different types of the TM. Contrary to previous discussions, type 2 TM can also occur in young patients.

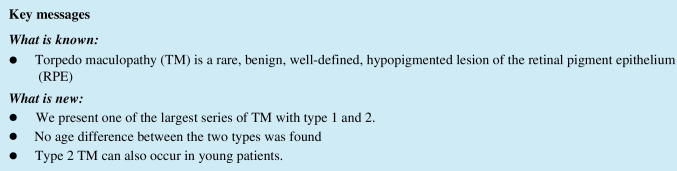

## Introduction

Torpedo maculopathy (TM) refers to a rare, benign, well-defined, hypopigmented, presumably congenital lesion of the retinal pigment epithelium (RPE), typically appearing as a “torpedo-shaped” structure with a wedge-shaped tail in the temporal macula along the horizontal raphe pointing towards the fovea [1, 2]. It is often presenting as an incidental unilateral finding [1].

The typical fundus lesion can be distinguished from other lesions, such as toxoplasma scar, traumatic injury, congenital hypertrophy of the RPE, or congenital RPE hypertrophy associated with Gardner syndrome [3].

The variably pigmented TM lesion, with rare foveal involvement, has been hypothesized to be the result of a developmental abnormality, including a defect in RPE development at the temporal fetal bulge [4], malformation of an emissary canal [1], or incomplete arcuate bundle differentiation [5]. However, the etiology of TM still remains unknown.

Based on optical coherence tomography (OCT), Wong et al. described two morphological phenotypes of TM. Type 1 TM is defined as outer retinal attenuation of the interdigitation zone and ellipsoid zone and eventually thinning of the outer nuclear layer. Outer retinal cavitation is not present. Type 2 TM is characterized by outer retinal degeneration (interdigitation zone and ellipsoid zone and thinning of the outer nuclear layer) and outer retinal cavitation with or without inner choroidal excavation [6]. In both types, the inner retina is normal and there is an increased signal transmission to the choroid. This classification has been extended by Tripathy et al. to propose type 3 TM, which does not strictly fall into either type 1 or type 2, and shows additional choroidal and inner retinal excavation along with outer retinal degeneration and retinal thinning [7]. Numerous reports have been added to the literature, but sample sizes have been relatively small and disjointed [8]. There is limited knowledge whether this classification is exhaustive in describing the spectrum of possible OCT morphologies inherent to this clinical entity [9]. Typically, TM is a stationary disorder. However, changes over time have also been discussed [10].

The aim of the present case series was to illustrate the morphological and clinical spectrum of TM lesions of the retina.

## Methods

The study adhered to the tenets of the Declaration of Helsinki and approval was obtained from the Ethics Committee of the Medical Faculty of the University of Tuebingen (600/2022BO2). Written informed consent was obtained from all patients or their legal guardians.

The search for electronic medical records of patients diagnosed with TM at the Eye Hospital of the University of Tuebingen identified eight patients between 2016 and 2022. The electronic records of these patients were reviewed and demographic information was recorded. All patients underwent detailed ophthalmological examinations including fundus imaging using Optos (Daytona, Optos PLC, UK), and optical coherence tomography (OCT) and fundus autofluorescence (FAF) imaging using Spectralis (Heidelberg Engineering, Heidelberg, Germany) or Optos (Daytona, Optos PLC, UK). Two patients had Swept-Source OCT angiography (PLEX Elite 9000, ZEISS, Jena, Deutschland), and two patients had perimetry (one with Octopus 900, 30–2, Haag-Streit Diagnostics, Wedel, Germany; the second with microperimetry, MAIA, CenterVue, San Jose, CA, USA).

## Results

**Fig. 1:**
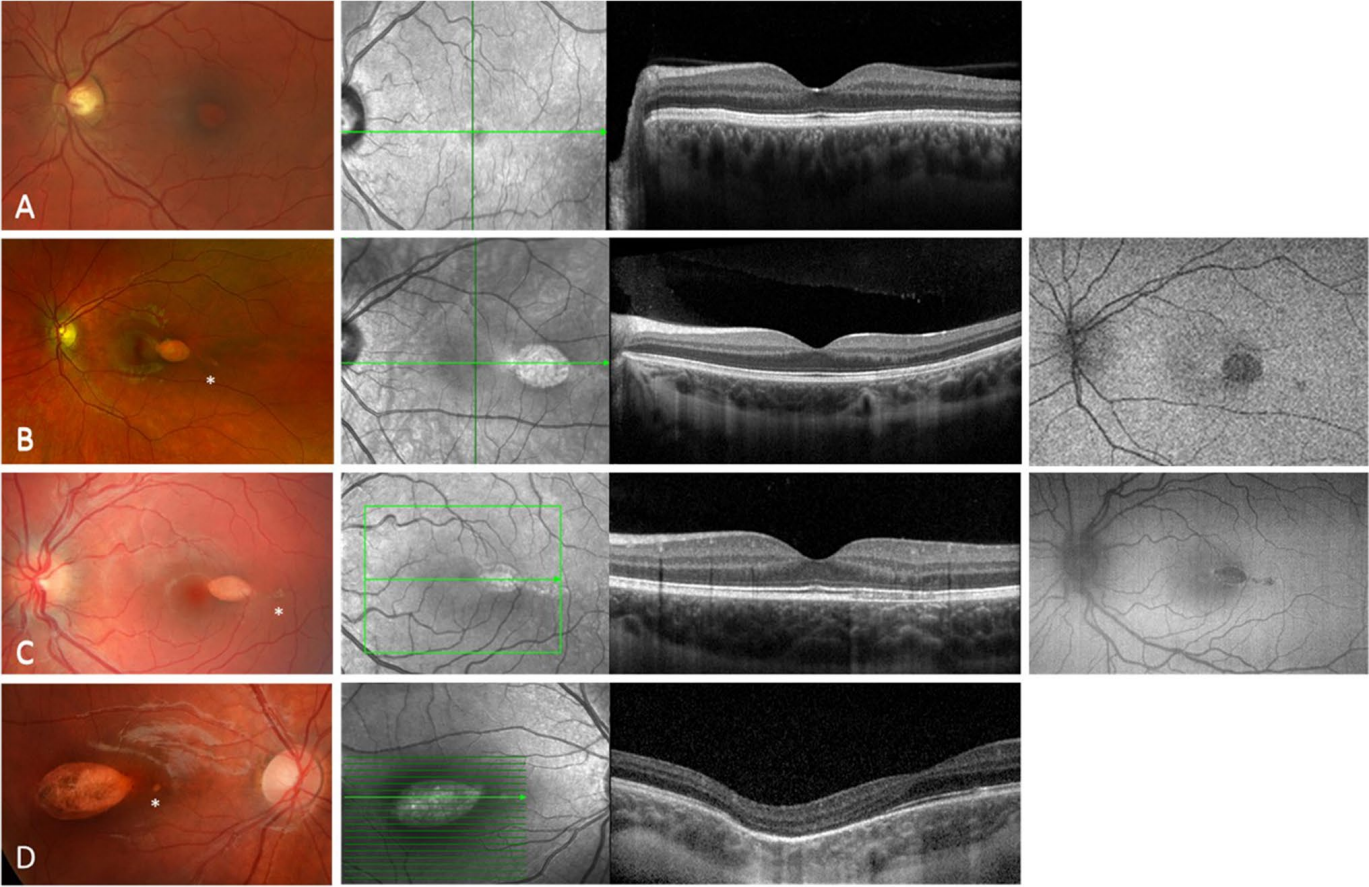
Color fundus photography, optical coherence tomography (OCT), and fundus autofluorescence (AF) imaging using Spectralis (Heidelberg Engineering, Heidelberg, Germany) or Optos (Daytona, Optos PLC, UK) of patients 1 to 4 (**A**–**D**) with type 1 torpedo maculopathy (TM). Note that all hypopigmented lesions are located temporal to the fovea (**A**–**D**) and can also be seen in the near infrared imaging (except for **A**). OCT images show normal inner retina in all eyes. In the area of TM lesion, attenuation of the interdigitation zone can be seen in mild cases (patients 1–3; **A**–**C**). Patient 4 (**D**) shows thinning of the outer nuclear layer and loss of ellipsoid and interdigitation zone and a small nasal satellite lesion (*). Patients 2 (**B**) and 3 (**C**) show a small temporal satellite lesion (*) and hypo-AF and hyper-AF boundaries (**C**)

**Fig. 2:**
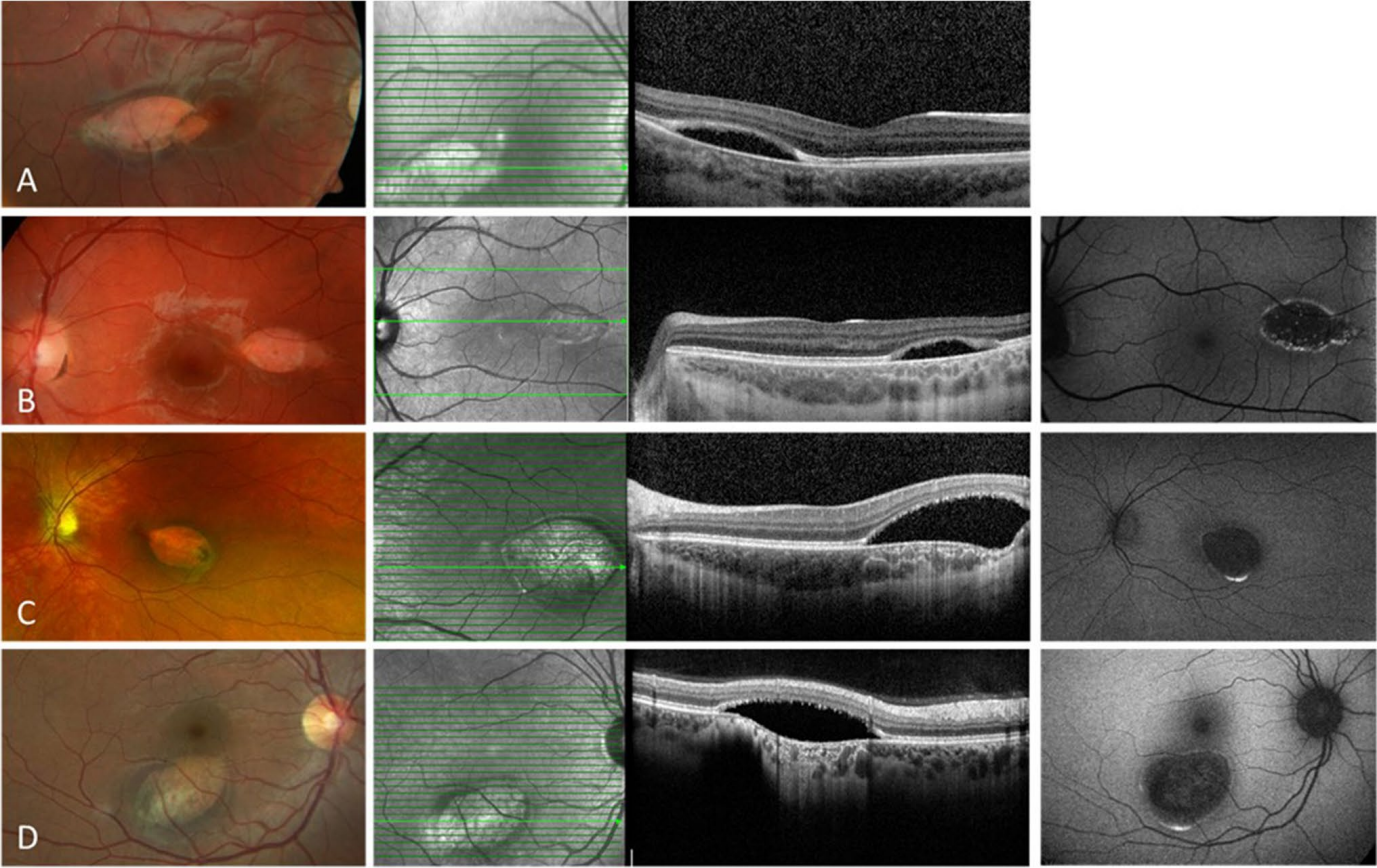
Color fundus photography, optical coherence tomography (OCT), and fundus autofluorescence (AF) imaging using Spectralis (Heidelberg Engineering, Heidelberg, Germany) or Optos (Daytona, Optos PLC, UK) of patients 5 to 8 (**A**–**D**) with type 2 torpedo maculopathy (TM). The hypopigmented lesions are located temporal to the fovea (**A**–**C**) except for patient 8 (**D**) with an inferior location. All lesions can be seen in the near infrared imaging (**A**–**D**). OCT images show normal inner retina in all eyes. All patients show thinning of the outer nuclear layer and loss of ellipsoid and interdigitation zone at the TM lesion with a subretinal cavitation/cleft. Patients 7 and 8 (**C**, **D**) additionally show hyperpigmentation with choroidal excavation. Patients 6–8 (**C**–**E**) show a hypo-AF with hyper-AF boundaries

Eight eyes diagnosed with TM were included in this case series. The average age of the patients (four males, four females) was 20.5 years (range 4–52 years). All cases were unilateral. All patients were asymptomatic on presentation and visual acuity in the affected eye was 1.0 decimal (Snellen equivalent 20/20). None of the patients had any associated systemic conditions. On color fundus photography, all lesions were hypopigmented with associated surrounding hyperpigmented RPE changes to varying degrees (Table 1). The lesions were located in the temporal retina on the horizontal axis, pointing towards the fovea, except for one patient with a lesion inferior to the fovea (patient 8) (Figs. 1 and 2). Patients 2 and 3 showed a flat temporal and patient 4 a flat nasal satellite lesion (Fig. 1B–D). Follow-up data are available for patients 1, 3, 5, and 7 over 6 to 20 months. The patients did not show any change of the TM lesion during the follow-up period.

**Table 1:** Patient data

ID	Age	Sex	Affected eye	VA decimal	Color fundus	Autofluo	OCT	Type
1	52	w	L	1.0	Temporal to fovea, hypopigmented		Minimal attenuation interdigitation zone	1
2	9	w	L	1.0	Temporal to fovea, hypopigmented	Hypo-AF	Minimal ONL thinning Attenuation interdigitation zone	1
3	7	m	L	1.0	Temporal to fovea, hypopigmented	Hypo-AF with hyper-AF border	Minimal ONL thinning Attenuation interdigitation zone	1
4	4	m	R	1.0	Temporal to fovea, hypopigmented with hyperpigmentation temporal		Thinning ONL Thinning ellipsoid zone Thinning interdigitation zone Inner choroidal excavation	1
5	5	w	R	1.0	Temporal to fovea, hypopigmented		Thinning ONL Loss ellipsoid zone Loss interdigitation zone Subretinal cleft/cavitation	2
6	11	m	L	1.0	Temporal to fovea, hypopigmented	Hypo-AF with hyper-AF border	Thinning ONL Loss ellipsoid zone Loss interdigitation zone Subretinal cleft/cavitation	2
7	11	m	L	1.0	Temporal to fovea, hypopigmented with hyperpigmentation temporal	Hypo-AF with inferior hyper-AF border	Thinning ONL Loss ellipsoid zone Loss interdigitation zone Subretinal cleft/cavitation Focal choroidal excavation temporal	2
8	39	w	R	1.0	Inferior, hypopigmented	Hypo-AF with hyper-AF border	Thinning ONL Loss ellipsoid zone Loss interdigitation zone Subretinal cleft/cavitation Focal choroidal excavation temporal	2

*R* right, *L* left, *VA* visual acuity, *OCT* optical coherence tomography, *ONL* outer nuclear layer, *AF* autofluorescence

OCT was performed in all patients (Figs. 1 and 2). The inner retina was normal in all eyes. In the area of TM lesion, attenuation of the interdigitation zone was seen in mild cases (patients 1–3). All other five patients had thinning of the outer nuclear layer and loss of ellipsoid zone and interdigitation zone at the TM lesion. Subretinal cavitation/cleft was noted in four cases (patients 5–8), while two cases (patients 7 and 8) presented with choroidal excavation. No patient had any sign of choroidal neovascularization (CNV). With regard to the classification according to Wong et al., we categorized the patients as follows: patients 1 to 4 type 1, patients 5 to 8 type 2 [6]. The average age at the presentation was 18 years (4, 7, 9, and 52 years) for patients 1 to 4 and 16.5 years (5, 11, 11, and 39 years) for patients 5 to 8.

FAF imaging using short wavelength blue light performed in five eyes (patients 2, 3, 6, 7, and 8) identified a hypo-autofluorescent (AF) lesion with hyper-AF boundaries in four eyes (patients 3, 6, 7, and 8). Fluorescein angiography was also performed in patient 8, revealing hyperfluorescence in the lesion due to a window defect created by the TM lesion. OCT angiography was performed in two cases (patients 3 and 8), showing a rarefied choriocapillaris. Two patients (7 and 8) had an unremarkable visual field test.

## Discussion

In our study, we present one of the largest case series of patients with TM. All cases exhibited a hypopigmented lesion at the posterior pole, mostly located immediately temporally adjacent to the fovea with a characteristic pointed tip. To date, more than 80 patients have been described in the literature, but those are mainly case reports [8]. The largest case series contain 5, 9, and 13 patients [1, 6, 11].

Although TM is classically described as a solitary oval lesion with variable pigmentation, satellite lesions can also occur [11], as observed in patients 2, 3, and 4 in our series. All satellite lesions reported to date have been located temporally and are usually small, round, and flat. However, in patient 4, there appears to be a satellite focus in the nasal region of the TM which has not been described before. OCT imaging reveals changes similar to those in the main TM lesion [11]. Bilateral cases have also been described [12].

Common features described for OCT scans include thinning of the outer nuclear layer, and attenuation or loss of the ellipsoid zone and interdigitation zone. Additional features, such as the presence of a subretinal cavitation, with or without inner choroidal excavation, have helped in classifying TM in types 1 and 2 [6]. Thereby, choroidal excavation along with a degeneration of the outer retina and retinal thinning in TM lesions were proposed to be described as type 3 by Tripathy et al. [7]. In our case series, we did not see type 3 TM [7]. Furthermore, we could not confirm the occurrence of the different types by age. Wong et al. concluded that type 1 tended to be younger than those with type 2 lesion [6]. However, this age estimation was based on the analysis of only 5 cases.

Kerwat et al. mentioned that, initially, the benign pathology of TM might start with a structurally normal retina with no fluid accumulation, and dysgenetic changes in the retinal pigment epithelium might lead to secondary accumulation of fluid over time [13]. The authors based their observations on 4 cases, the oldest of which had subretinal fluid [13]. In our case series, mean age and age range were similar for type 1 and type 2 TM and we also observed type 2 TM in younger age. Similarly, Mesnard et al. showed type 2 TM in a 23-month-old baby [14]. The facts that TM typically is an incidental finding due to the lack of symptoms and is mostly considered a stationary condition may have contributed that long-term follow-ups including imaging studies have not been published so far.

The underlying cause of TM is still unknown. A literature review conducted by Williams et al. showed that TM is mostly considered congenital because the lesions are near the temporal macula in the majority (77) of the cases and age distribution was 6 months to 73 years (mean 24.2 years) [8]. In our case series, the location of the lesion and the age of the patients are in line with the above-mentioned findings. Due to the consistent location of TM, the most commonly accepted hypothesis for the etiology is a persistent defect in the development of the RPE in the fetal temporal bulge [4]. Two other main theories are that TM might be caused by a developmental defect within the nerve fiber layer at the horizontal raphe [5], or that the lesion may be related to dysmorphia of the emissary canal of the long posterior ciliary artery and nerve [1].

Fluorescein angiographic studies may show a window defect. The transmission defect was also present in our case, consistent with an atrophied RPE. Therefore, autofluorescence imaging may show reduced or absent autofluorescence, which may indicate not only a loss of RPE pigmentation but also a general loss of RPE. A hyperautofluorescent rim may be present in TM.

Functional tests with perimetry/microperimetry may reveal reduced sensitivity or scotoma in the area of the lesion due to reduced retinal sensitivity [15–17]. However, we could not detect any visual field defect in two tested patients. Also, Ding et al. showed that microperimetry visual field was basically normal in a multimodal imaging case report [3].

Although patients with TM are asymptomatic and experience a benign and non-progressive disease course, the occurrence of choroidal neovascularization (CNV) in the presence of TM has been described [18–20]. Venkatesh et al. suggested that CNV development in TM can occur in patients with type 2 or 3 findings on OCT [11]. The patients in our case series with significant hyperpigmentation temporal to the TM also have choroidal excavation.

The limitations of this study are the retrospective study design with no or short follow-up due to the benign character of TM. Furthermore, standard multimodal imaging protocol was not performed in all patients.

In conclusion, we present one of the largest series of TM with types 1 and 2. We did not observe any type 3 lesion. Contrary to previous reports, we did not find an age difference between the different types, as both types were young and similar in age. Therefore, contrary to the previous opinion, we conclude that type 2 TM can also occur in young patients. Long-term studies are needed to elucidate whether the lesions show any progress from a type 1 to 2 lesion with age.

## References

[1] Golchet PR, Jampol LM, Mathura JR, Daily MJ (2010). Torpedo maculopathy. Br J Ophthalmol.

[2] Trevino R, Kiani S, Raveendranathan P (2014). The expanding clinical spectrum of torpedo maculopathy. Optom Vis Sci off publication Am Acad Optom.

[3] Ding Y, Yao B, Ye H, Yu Y (2019). Multimodal imaging of torpedo maculopathy in a Chinese woman: a case report. BMC Ophthalmol.

[4] Shields CL, Guzman JM, Shapiro MJ, Fogel LE, Shields JA (2010) Torpedo maculopathy at the site of the fetal “bulge”. Archiv Ophthal (Chicago, Ill. 1960) 128:499–501 10.1001/archophthalmol.2010.29 20385950

[5] Pian D, Ferrucci S, Anderson SF, Wu C (2003). Paramacular coloboma. Optom Vis Sci off Publication Am Acad Optom.

[6] Wong EN, Fraser-Bell S, Hunyor AP, Chen FK (2015). Novel optical coherence tomography classification of torpedo maculopathy. Clin Experiment Ophthalmol.

[7] Tripathy K, Sarma B, Mazumdar S (2018). Commentary: Inner retinal excavation in torpedo maculopathy and proposed type 3 lesions in optical coherence tomography. Indian J Ophthalmol.

[8] Williams PJ, Salek S, Prinzi RA, Bergstrom C, Hubbard GB (2019). Distribution patterns of torpedo maculopathy: further evidence of a congenital retinal nerve fiber layer-driven etiology. Saudi J Ophthalmol Off J Saudi Ophthal Soc.

[9] Light JG, Alvin Liu TY (2020). A novel phenotype of torpedo maculopathy on spectral-domain optical coherence tomography. Am J Ophthalmol Case Rep.

[10] Baker D, Nur I (2022) Letter to the editor: Torpedo maculopathy: a case series - insights into basic pathology. Eur J Ophthalmol 32:NP311-NP312 10.1177/1120672120957595 PMC8777319 32933313

[11] Venkatesh R, Jain K, Pereira A, Thirumalesh YNK (2020). Torpedo retinopathy. J Ophthalmic Vis Res.

[12] Richez F, Gueudry J, Brasseur G, Muraine M (2010). Maculopathie en torpille bilatérale. Journal francais d’ophtalmologie.

[13] Kerwat D, Jamall O, Antonakis S, Almeida GC (2021) Torpedo maculopathy: a case series - insights into basic pathology. Eur J Ophthalmol 31:NP35-NP39 10.1177/1120672120905313 32037872

[14] Mesnard C, Benzekri R, Chassery M, Ventura E, Merle H (2020). Ocular manifestations in congenital Zika syndrome: about a case of torpedo maculopathy. Am J Ophthalmol Case Rep.

[15] Buzzonetti L, Petroni S, Catena G, Iarossi G (2015). Optical coherence tomography and electrophysiological findings in torpedo maculopathy. Doc Ophthalmol Adv Ophthalmol.

[16] Su Y, Gurwood AS (2010). Neurosensory retinal detachment secondary to torpedo maculopathy. Optometry (St. Louis, Mo.).

[17] Sanabria MR, Coco RM, Sanchidrian M (2008). Oct findings in torpedo maculopathy. Retin Cases Br Rep.

[18] Shirley K, O’Neill M, Gamble R, Ramsey A, McLoone E (2018). Torpedo maculopathy: disease spectrum and associated choroidal neovascularisation in a paediatric population. Eye (Lond).

[19] Parodi MB, Romano F, Montagna M, Albertini GC, Pierro L, Arrigo A, Bandello F (2018). Choroidal neovascularization in torpedo maculopathy assessed on optical coherence tomography angiography. Ophthalmic Surg Lasers Imaging Retina.

[20] Jurjevic D, Böni C, Barthelmes D, Fasler K, Becker M, Michels S, Stemmle J, Herbort C, Zweifel SA (2017). Torpedo-Makulopathie mit choroidaler Neovaskularisation. Klin Monatsbl Augenheilkd.

